# The Relevance of Plant-Derived Se Compounds to Human Health in the SARS-CoV-2 (COVID-19) Pandemic Era

**DOI:** 10.3390/antiox10071031

**Published:** 2021-06-25

**Authors:** Leonardo Warzea Lima, Serenella Nardi, Veronica Santoro, Michela Schiavon

**Affiliations:** 1 Biology Department, Colorado State University, Fort Collins, CO 80523, USA; leolima@rams.colostate.edu; 2 Department of Agronomy, Food, Natural Resources, Animals and Environment (DAFNAE), University of Padova, Viale dell’Università 16, 35020 Legnaro, PD, Italy; serenella.nardi@unipd.it; 3 Department of Agricultural, Forest and Food Sciences (DISAFA), University of Turin, Via Leonardo da Vinci, 44, 10095 Grugliasco, TO, Italy; veronica.santoro@unito.it

**Keywords:** plant-selenium compounds, selenite, organic Se, selenoproteins, COVID-19

## Abstract

Dietary selenium (Se)-compounds accumulated in plants are essential for human metabolism and normal physiological processes. Inorganic and organic Se species can be readily absorbed by the human body, but are metabolized differently and thus exhibit distinct mechanisms of action. They can act as antioxidants or serve as a source of Se for the synthesis of selenoproteins. Selenocysteine, in particular, is incorporated at the catalytic center of these proteins through a specific insertion mechanism and, due to its electronic features, enhances their catalytic activity against biological oxidants. Selenite and other Se-organic compounds may also act as direct antioxidants in cells due to their strong nucleophilic properties. In addition, Se-amino acids are more easily subjected to oxidation than the corresponding thiols/thioethers and can bind redox-active metal ions. Adequate Se intake aids in preventing several metabolic disorders and affords protection against viral infections. At present, an epidemic caused by a novel coronavirus (SARS-CoV-2) threatens human health across several countries and impacts the global economy. Therefore, Se-supplementation could be a complementary treatment to vaccines and pharmacological drugs to reduce the viral load, mutation frequency, and enhance the immune system of populations with low Se intake in the diet.

## 1. Introduction

Selenium (Se), as an intrinsic component of essential selenoproteins, is required in traces for preserving the optimal health and balanced metabolism of mammals [1]. The twenty-five selenoproteins discovered in humans to date play central roles in the cell redox status and antioxidant processes, hormone metabolism, the immune system, and cardiovascular and reproductive functioning maintenance [1,2,3,4,5,6,7,8]. Among these proteins, it is worth mentioning thioredoxin reductases (TrxRs), implied in the control of thyroid metabolism, selenoprotein P (SelP), the most abundant selenoprotein in plasma secreted by the liver and supplying selenium to all other tissues, and Selenoprotein-S, which regulates the inflammatory cytokines. In addition, glutathione peroxidases (GPx), a well-documented category of reactive oxygen species (ROS) scavenging enzymes, catalyze the destruction of hydrogen peroxide and other hydroperoxides with the help of glutathione, and reduce phospholipid hydroperoxide in cell membranes [9,10]. A depletion of selenoproteins due to inadequate dietary Se intake might result in lower resistance against oxidative stress and higher incidence of severe disease outcomes, such as skeletal myopathy and cardiomyopathy, thyroid disorders, and reduced male fertility [2,11,12,13,14,15]. Se deficiency has also been associated with an increased susceptibility of individuals to cancer development and RNA viral infections [10,15,16]. Keshan disease, for instance, is a potentially lethal childhood cardiomyopathy which is prevalent in northeast China. The associated myocardial necrosis, is caused by a merged condition of Se deficiency and secondary viral infection associated with Coxsackievirus [17].

Se is recognized as an ambivalent element despite its essentiality to humans and other organisms [1,18,19]. Indeed, a relatively narrow window exists between Se intakes that determine benefit or toxicity. In areas where Se-related diseases are endemic, populations frequently ingest a daily Se dietary intake below 15 μg/day, which is far lower than the recommended dietary allowance (RDA) of Se for adults set at 55 μg/day [20]. On the other hand, the steady intake of over 400 μg/day of Se might pose a threat for consumers, who may suffer from chronic Se toxicity, a condition also termed selenosis or alkali disease [21].

Although severe Se deficiency is a rare condition, there is extensive evidence that suboptimal Se status is relatively common across the world [22,23]. Adequate or supranutritional Se supplementation to vulnerable populations through Se-enriched food has been proposed as a natural approach to reducing metabolic disorders, preventing certain forms of cancer (e.g., gastrointestinal and prostate cancer), and enhancing the immune system [6,15,18,24,25]. However, the role of Se compounds in cancer prevention and therapy is under debate due to the contrasting results achieved from clinical, epidemiologic, and laboratory trials [26,27]. On the other hand, the positive effects of Se in combating viral infections have been consistently reported [15,16,28].

Although plants do not need Se, they represent a major source of this element for humans and animals [29,30]. They can take up Se, primarily as inorganic Se, and convert it to organic forms, mainly Se-amino acids (e.g., selenocysteine, selenomethionine) that serve as direct antioxidants to consumers [31,32]. The amino acid selenocysteine (SeCys) is also an inherent component of human selenoproteins, being specifically incorporated at their catalytic site [2]. Due to the redox properties of Se, selenoproteins exhibit excellent antioxidant activity in biological systems. Certain plants, namely Se hyperaccumulators, possess a supplementary metabolic pathway that allows them to generate elevated amounts of methylselenocysteine (MeSeCys) [19]. Elevated concentrations of such a compound are associated with chemoprevention and, following oxidative decomposition by β-lyases, produce methylselenenol, which is the simplest organic selenium compound with pharmacological action [33,34,35]. Several plants, especially members of Brassicaceae, Capparidaceae, and Euphorbiaceae, can synthesize additional beneficial Se-compounds, such as seleno-glucosinolates [36,37].

In this review, we aim to provide an overview of the Se-compounds that are generated in plants by considering the metabolic processes that lead to their synthesis, the chemical features that make them unique in human biology, and their role as direct and indirect antioxidants in cells, and to support the importance of their consumption as a complementary treatment to vaccines and pharmacological medicines in the context of the pandemic caused by the severe acute respiratory syndrome coronavirus 2 (SARS-CoV-2), the infectious agent causing the Coronavirus Disease 2019 (COVID-19). SARS-CoV-2 is a coronavirus not previously observed in humans. It has a positive-sense single-stranded RNA (ssRNA+) as a genome (26–32 kb) and targets cells expressing the angiotensin-converting enzyme two receptors (ACE2) (e.g., airway epithelial cells, alveolar epithelial cells, vascular endothelial cells, and macrophages in the lung, as well as myocardial and renal cells), thus causing principally respiratory and gastrointestinal pathogenic conditions, and aggressive inflammatory responses [10,15]. Beyond posing a severe threat to health at the global level, COVID-19 disease also provokes tremendous consequences on the world economy.

## 2. Selenium in Plants

### 2.1. Selenium in the Soil and Uptake by Plants

Plants are the primary source of dietary Se for humans and animals. However, the uptake and accumulation of Se by plants are not simple processes. This is due to the fact that Se is not considered to be a nutrient for these organisms, and its bioavailability to plants is governed by complex physicochemical processes occurring in the soil. To better understand the role of plants as good sources of dietary Se, two questions must hence be first answered: what are the main origins of Se, and what is the fate of this metalloid in the soil that may further affect the capacity of the plant to accumulate it?

The presence of Se in the soil can vary greatly due to different anthropogenic activities, mainly mining and agriculture. However, Se naturally and continuously cycles through the environment. Its concentration in the soil is governed by a multitude of processes, including precipitation (via atmospheric Se deposition) and Se speciation, and the soil properties such as pH, redox potential, structure, and organic matter content and composition [38,39,40]. There is no defined point of entry of Se to the environment, however the literature agrees the Se variation in soils is driven by its unequal distribution between sources and sinks [41,42].

Soil is formed by parenting rock weathering, naturally composed of different trace elements and minerals, including Se. This metalloid is typically found at high concentrations in clay-rich sedimentary rocks such as shale, as an example, formed by volcanic activity [39,40]. Furthermore, the concentration of Se tends to be naturally high in other soil formations where the sulfur (S) concentration is also elevated. It is important to note the adsorption of Se species to soil is correlated with the oxidation state of the atom and the pH of the soil, with increased adsorption to soil particles with decreasing pH values, predominantly below pH 6 [38].

While the weathering of parent rocks can be considered one of the primary natural sources of Se, different atmospheric and geogenic sources of Se to soil have been extensively studied [42]. It is estimated that a minimum of 13,000 tons of Se is cycled in the troposphere yearly [41,43] from different natural and anthropogenic sources. Volcanic activity [39,44] and industrial processes, such as waste from the crude oil refining process and fossil fuel combustion, are the principal source of atmospheric Se [39,41].

In the soil, Se can be found at different oxidation states and under organic or inorganic forms. The oxyanions selenate (VI), as SeO_4_^2−^, and selenite (IV), mainly as HSeO_3_^−^ and SeO_3_^2−^, are commonly found in drained soil at pH values between 4 and 9. These forms are soluble, and thus largely bioavailable to plants; however, their retention by soil particles increases when the pH decreases. The most reduced inorganic forms of Se that occur in natural environments are elemental Se (Se(0)) and selenides, including hydrogen selenide and different metallic selenides, produced by microbial activity [45]. However, these latter Se species are insoluble and not bioavailable. Generally, the bioavailability of Se increases in more oxidizing environments, where selenate ions tend to be highly soluble and mobile in aerated, alkaline, and oxidized soils. Selenite, instead, predominates in more acidic and reducing environments [46,47].

Organic matter (OM) also plays a role in the retention, bioavailability, and mobilization of Se in the soil. The OM can form colloids with Se and increase its retention, and some studies suggest the OM-Se colloids might correspond up to 50% of the total soil Se in seleniferous areas [45,46]. The immobilization of Se by the soil OM is more prominent when the Se levels are relatively low; however, the type of soil and the composition of the OM is more relevant to Se mobilization than its concentration [46].

The most important aspect to be considered when studying Se accumulation by plants is the chemical similarity between Se and S. These two elements can be found in group 16 of the oxygen family (the chalcogens) in the periodic table. Their ionic radius, redox potentials, and electronegativity are similar [48]. Selenate, the most common form of Se taken up by plants in soils, is taken up by the root system via sulfate transporters, *Sultr* [49,50,51], while selenite uptake is mediated by phosphate and silicon transporters [52,53,54]. Sulfate transporters were first characterized in Se resistant mutants of *Arabidopsis thaliana*, *Sel1-8*, and *Sel-11* (mutations in the *Sutr1;2* coding sequence), and *Sel1-9* (T-DNA insertion in the *Sultr1;2* promoter), [55,56]. Four groups of sulfate transporters have been identified in plants and are responsible for the uptake and translocation of Se. *Sultr 1;1* and *Sultr1;2* are high affinity H^+^ co-transporters localized at the root hairs, cortex, and epidermis [57]; *Sultr 2;1* is expressed in the xylem parenchyma and pericycle, while *Sultr 2;2* is present in the phloem and bundle sheath cells [58]; *Sultr 3;1* is a chloroplast transporter [59]; and *Sultr 4;1* and *Sultr 4;2* are efflux transporters found in the tonoplast [49].

### 2.2. Selenium Metabolism in Plants

The Se metabolism in plants is complex, and the misincorporation of Se to the amino acid Cys, via S replacement, causes toxicity that manifests at Se tissues concentrations variably depending on the plant species (in most cases, from a few μg Se/mg^−1^ DW to 100 μg Se/mg^−1^ DW). The degree of Se toxicity mainly depends on the plant capacity to prevent the occurrence of SeCys and SeMet insertion into proteins. Plants can in fact be organized into different groups according to their capacity to accumulate and tolerate Se. Certain species evolved various mechanisms to avoid Se toxicity by synthesizing organic and less toxic chemical forms of Se. Crops and most plant species cannot accumulate large amounts of Se and are called non-accumulators [51]. The Se concentrations in their tissues is usually below 100 μg Se/mg^−1^ DW. Certain species, *Brassica juncea* (Brown mustard) and *Brassica napus* (Canola), for example, can accumulate up to 1000 μg Se/mg^−1^ DW while growing in a natural environment, without suffering any symptoms of stress, and are classified as secondary accumulators. Their Se levels are directly related to the Se availability in the soil, so they can also be referred to as Se-indicators [19]. Some species can accumulate Se concentration above 1000 μg Se/mg^−1^ DW and are termed Se-hyperaccumulators. These include plants from the genera Stanleya (Brassicaceae) and Astragalus (Fabaceae), among others, and have evolved specific biosynthetic pathways to methylate and volatilize the amino acid SeCys [51].

After the uptake, Se, either in the form of selenate or selenite, is translocated from the roots to the leaves, where it is assimilated in the chloroplasts. The first step in its assimilation pathway is carried out by the enzyme ATP-sulfurylase, responsible for coupling selenate to ATP, forming adenosine 5’-phosphoselenate (APSe) [60]. APSe can be utilized in a parallel metabolic branch to produce PAPSe (3′-phosphoadenosine 5′-phosphoselenate) via the enzyme adenosine-5′-phosphosulfate kinase (APSK), analogous to the PAPS (3′-phosphoadenosine 5′-phosphosulfate), an essential substrate for the S/Se secondary metabolites synthesis, such as glucosinolates (GLS). The APSe is further reduced to selenite via the APS reductase enzyme [50,51,61]. Next, selenite is reduced to selenide (Se^2−^) by the enzyme sulfite reductase (SiR) [51]. Alternatively, this process can also occur by a series of reactions between selenite and reduced glutathione (GSH), utilizing the enzyme glutathione reductase (GR) to synthesize Se^2−^ [50,51,61]. The next step couples Se^2−^ to O-acetylserine (OAS) via the enzyme cysteine synthase (CS) to form the first Se amino acid, SeCys. This step involves two enzymes working together in a series of processes. Serine acetyltransferase (SAT) synthesizes the OAS, while the O-acetylserine thiol-lyase (OAS lyase) couples the OAS to Se^2−^, forming the SeCys. The amino acid SeCys can also be synthesized directly from Se IV by the action of seleno-methyltransferase (SeMT) [51,61].

SeCys can have different metabolic fates in cells. Non-hyperaccumulator plants would either incorporate this amino acid into proteins or continue with the cascade of reductions to form another amino acid, SeMet. SeCys can be reduced to form SeCystathionine, via the enzyme cystathionine g-synthase (CGS), which couples the SeCys amino acid to O-phosphohomoserine (OPH). SeCystathionine can now be reduced to SeHomocysteine (SeHCys) by the enzyme cystathionine β-lyase (CBL); this process still happens in plastids. SeHCys leaves the plastid and is reduced to the amino acid SeMet in the cytosol by the enzyme methionine synthase (MS) [50,51]. Alternatively, Se-hyperaccumulator plants can methylate SeCys via the enzyme SeCys methyltransferase (SMT) to form MeSeCys (methylselenocysteine) the main form of organic Se found in these plants, and also one of the most beneficial forms of Se to consumers [19,51,62].

**Figure 1:**
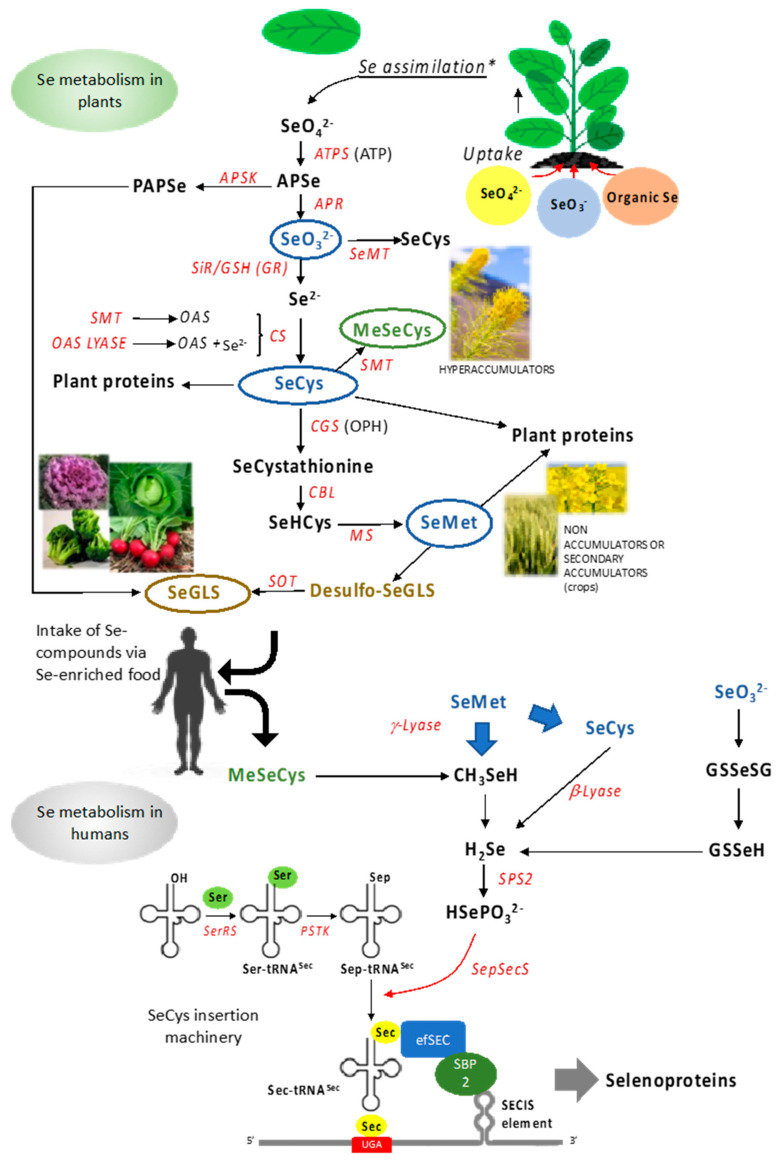
Flux of Se compounds from plants to humans. Top: metabolic pathway of Se in plants. Bottom: assimilation of Se compounds in human cells and specific mechanism of SeCys insertion in selenoproteins. In blue are compounds produced by all plants, in green are those specifically produced by Se-hyperaccumulators and in yellow are those generated in plant species belonging to Brassicaceae, Capparidaceae, and Euphorbiaceae families. Enzymes are indicated in red. All acronimous are given in the text.

Some plants of the Brassicales order (broccoli, cabbage, mustard, and cauliflower, among others) can produce an extensive group of more than 130 aliphatic, indolic, or aromatic secondary metabolites, namely glucosinolates (GLS), synthesized in different vascular tissues [63]. GLS are sulfur and nitrogen compounds that use different amino acids as precursors, including Met in the case of the aliphatic GLS. These secondary metabolites are used by plants as a defense mechanism against herbivores and different pathogens, and afford benefits to consumers. Each subtype of GLS has its precursors and is synthesized independently; however, all biosynthetic pathways follow the same general steps in the following order: side-chain elongation, formation of core molecule structure, and secondary modification [64,65]. SeMet can be used as a precursor of aliphatic GLS in place of Met, and the resulting (methylseleno) glucosinolates, as well as their Se-containing aglycons, are supposed to possess superior bioactivity as anticancer and antimicrobial agents compared to GLS [36,37]. Figure 1 depicts Se metabolic pathways in plants.

Inorganic Se and organic Se forms produced by plants have been used in medical and pharmacological trials to assay their benefits on health. In more recent years, the advent of nanotechnology has allowed testing the effects of Se also in the form of nanoparticles, of size lower than 100 nm, especially for drug delivery.

SeNPs produced via laser ablation from Se pellets (~2mm diameters) in combination with dionized water, showed to have powerful antioxidant and anti-carcinogenic properties [66]. Tf-SeNPs, i.e., SeNPS conjugated with transferrin (Tf), chemically produced utilizing selenium dioxide (Na_2_SeO_3_) as a source of Se, also proved to be anticarcinogenic [67]. Interestingly, SeNPS are also known to have antimicrobial activity (Geoffrion et al, 2020; [66,68,69,70]. It is known that SeNPS, produced from Na_2_SeO_3_ and Arbidol (anti-viral agent), exhibt antiviral activities against H1N1 when administered in combination with the mentioned drug, by inhibiting the viruses of entering cells [71], showing great potential to be used against the novel SARS-CoV-2.

Beyond medical applications, nanoparticles can be applied in fields such as crop production, the food industry, pharmaceuticals, and medicine [72]. SeNPs, in particular, have attracted attention due to their low toxicity, biocompatibility, low-cost and simple reduction methods, and ability to be synthesized via UV radiation, hydrothermal techniques, or via cleaner and environmentally friendly methods of biogenic synthesis using a diverse of plant material, including leaf, seed, and fruit extracts [73].

### 2.3. Selenium Accumulation in Food Crops

Selenium in crop food is directly related to the level of Se found in the edible parts of crops, which is affected by its concentration and bioavailability in the soil and water. Several non-agricultural areas worldwide are known to contain very high Se concentration in their soil (seleniferous), including San Joaquin Valley in California, USA [74,75]; Wyoming, USA [76]; Hubei, China [77]; and Punjab, India [78]. However, low-Se areas are more commonly documented, and Se deficiency is estimated to affect 1 billion people worldwide [79].

Practices of biofortification can be adopted to increase the levels of Se in crops, to overcome the low dietary Se intake by the population living in low-Se areas [80]. The most direct way to increase the Se levels in the soil is the application of Se through inorganic and organic fertilizers. However, physicochemical properties of the soil can pose a challenge to the proper fortification of crops via soil fertilizers so other strategies, such as foliar application of Se, can be alternatively utilized.

Different reports support the evidence of positive health effects of GLS on human health, and advise the regular consumption of cruciferous vegetables such as broccoli (*Brassica oleracea* L. var. Italica) to reduce the risk of different forms of cancer and myocardial infarction [81]. It is suggested that GLS might have promising applications for other areas of medicine, including the potential against viral infections, considering the protective nature of these compounds to plants [81].

Interestingly, broccoli and forage rape, *Brassica napus* L., supplemented with sodium selenate, can synthesize selenoglucosinolates (SeGLS) by utilizing the analog amino acid SeMet as an aliphatic GLS precursor [36]. Three different forms of GLS were identified using liquid chromatography-mass spectrometry (LC-MS), described as glucoselenoiberverin, glucoselenoerucin, and glucoselenoberteroin. Other studies identified the incorporation of Se to other GLS compounds, including 2-phenylethylglucosinolate in roots of *Nasturtium officinale* [82] 3-butenylselenoglucosinolate in *Stanleya pinnata*, Prince’s Plume plants grown on hydroponics supplied with high Se concentration (grown for 21 days at a 100 ppm solution of Na_2_SeO_3_) [83]. Additional forms of SeGLS include glucoselenoraphanin and glucoselenoerucin in broccoli, glucoselenoiberverin in cauliflower and, in a more recent study, *Brassica oleracea* L. var. botrytis, and finally glucoselenonasturtiin, glucoselenoerucin, and glucoselenoberteroin in forage rape roots [37]. It was demonstrated in the past that the consumption of broccoli enriched with Se induced beneficial immune responses to antigens [84].

In the past fifteen years, several crop species were biofortified with Se on the field or via greenhouse experiments, where different sources of Se, as well as application methods, were studied. Organic forms of Se, including the amino acids SeMet and SeCys, were identified in corn (*Zea Mays* L.) grains after supplementation with sodium selenite via fertigation, utilizing 200 g of Se ha^−1^ [29]. Other cereal species were also studied due to their nutritional and economic importance. As an example, another study reported the presence of SeMet in mature bread wheat (*Triticum aestivum*) L. grains, and durum wheat grains, *Triticum durum* Desf., after soil and foliar application using either sodium selenate or sodium selenite, 4, 20, and 100 g of Se ha^−1^ [85]. Interestingly, the amino acid SeMet was found in all samples analyzed, regardless of the form of Se or mode of application. Another study reported similar results, where SeMet was determined in durum wheat grains after foliar spray in the field, using 0, 10, 20, and 40 g ha^−1^ of sodium selenate or sodium selenite [86].

The Se biofortification of legumes, bulb, and root plants and other relevant crops were also extensively analyzed in recent years. Chickpea (*Cicer arietinum* L.) grains, supplemented with sodium selenate or sodium selenite via foliar spray in the field, using a range of 0, 10, 20, and 40 g of Se ha^−1^, incorporated > 70%, of organic SeMet [87]. Soybean (*Glycine max* L.) accumulated SeMet and SeCys, after supplementation with sodium selenite of 0.9 mg of Se kg^−1^ of soil in a greenhouse experiment [88].

Although the amino acids SeMet and SeCys are more commonly found in Se biofortified crops, other distinct organic forms of Se were reported in the literature. Carrot (*Daucus carota* L.), produced SeMet and gamma-glutamyl-selenomethyl-selenocysteine (γ-glutamyl-SeMet-SeCys) after foliar application with sodium selenate or sodium selenite at 10 and 100 µg of Se mL^−1^, in a greenhouse experiment [89]. Broccoli and carrot grown on field-installed lysimeters, containing soil treated with *Stanleya pinnata* (selenium hyperaccumulator) powdered plant material, with a concentration of 700 µg of Se g^−1^ of DW, showed around 7% of MeSeCys among the total soluble seleno compounds in the broccoli florets and carrot roots [90].

Realistically, the implementation of Se-enriched fertilizers can be an expensive process for producers in low-Se areas. Alternatively, consumers can find good natural Se-enriched food, such as the Brazil nut (*Bertholletia excelsa* H.B.K.), known for having relatively high Se levels compared to any other plant-based food, and found to contain mainly organic C-Se-C, possibly SeMet, MetSeCys, or Se-lanthionine [91,92].

## 3. Antioxidant Properties of Plant Se-Compounds

### 3.1. Inorganic and Organic Se Species Functioning as Antioxidants and Immune Agents

**Figure 2:**
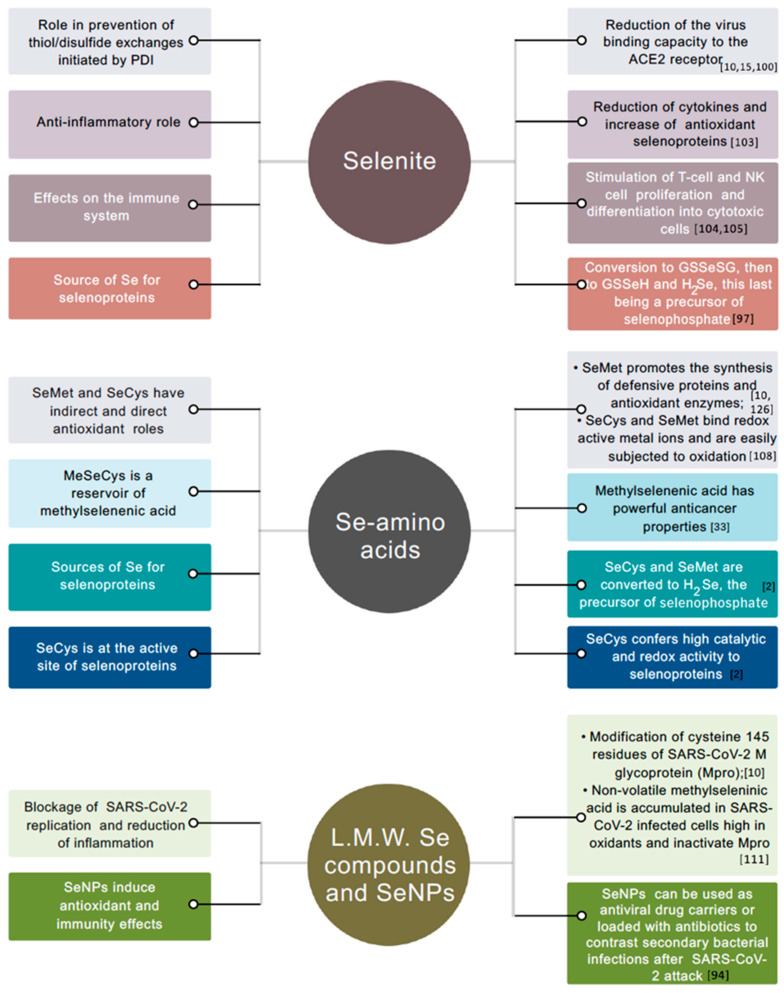
Left: principal roles of selenite, Se-amino acids, low molecular weight (L.M.W.) Se compounds, and seleno nanoparticles (SeNPs) in immunity and antioxidant processes in human cells. Right: mechanisms of action, either hypothesized or ascertained. The same color of the squared panels indicates association between role and mechanism of action.

There is broad evidence that inorganic and organic Se compounds, as well as Se-nanoparticles (SeNPs), have direct beneficial roles in mammals for a number of degenerative diseases and viral infections [1,28,93,94]. Inorganic and organic Se species, particularly, can be promptly absorbed from the gastrointestinal tract of the human body into the bloodstream, but are metabolized differently and exhibit distinct mechanisms of action in a variety of cellular processes [95,96] (Figure 2). Once in the bloodstream, selenite and selenate can bind to plasma proteins ([97], references therein). However, only limited selenite is incorporated in them. Otherwise, selenite remains free in the plasma, or is taken up and metabolized inside erythrocytes with GSH-mediated production of hydrogen selenide ions (HSe^−^) that are quicky effluxed, conveyed to the liver, and return to the plasma in the form of selenoproteins. In addition, the nonenzymatic reaction of selenite with GSH and Cys in plasma leads to the production of GS-Se-SG and Cys-Se-Cys species that may be potentially involved in the translocation to the liver. Conversely, selenate is reduced to selenite by plasma, and is assumed to be translocated from the bloodstream to the liver in its unmetabolized form [97].

The different biological activities of inorganic Se forms are associated with their electronic features. Only selenite has redox activity that aids in ameliorating cell viral infections, such as those caused by RNA viruses H1N1 and, possibly, SARS-CoV-2, to result in a better recovery and survival rate [98,99]. Similar to other coronaviruses, SARS-CoV-2 is a positive-stranded RNA virus with crown-like spike proteins on its surface. The large RNA genome encodes four major structural proteins: the spike (S) glycoprotein, nucleocapsid (N) protein, membrane (M) protein, and the envelope (E) protein, each of which is vital for the viral particle. The S1 domain of the virus glycoprotein binds the angiotensin-converting enzyme 2 (ACE2) entry receptor at the epithelial cells of the host nose, mouth, and lungs [100]. Then, the S2 domain of the same protein fuses with the host cell membrane, and the virus enters the cytosol via endocytosis [101]. The receptor-binding domain of the viral spike proteins and ACE2 has several cysteine residues, and a recent study provides evidence that the binding affinity is affected when their disulfide linkages are reduced to thiol groups [102]. Protein disulfide isomerases (PDIs) are redox enzymes that regulate the thiol–disulfide balance on the interactions between SARS-CoV/CoV-2 spike proteins and ACE2. Selenite prevents thiol/disulfide exchanges initiated by PDI during the attachment of viral glycoproteins to the host cell membranes, thus limiting the virus capacity to enter the host cells [6,99].

Beyond this, selenite has a recognized anti-inflammatory role that might help the recovery of individuals affected by viral infections such as COVID-19 and by other pathogenic conditions, such as cancer. Increasing selenite concentrations applied to human hepatoma cell lines, for instance, are reported to correlate with the reduction in pro-inflammatory cytokines (IL-6, IL-8, and IL-17) and increase the Se concentration in the protein fraction and the expression of antioxidant selenoproteins (e.g., GPx1, SELK) [103]. The anticancer properties of selenite and other Se compounds (selenate, SeMet, and SeCys) against certain forms of cancer are well ascertained. However, selenite supplementation apparently exerted better anticancer effects in some studies [58].

Selenite supplementation also has effects on the immune system, as reported in several trials. Se in the form of sodium selenite has been found to stimulate T-cell proliferation and promote innate immune-system functions [104]. Similarly, the administration of 200 μg/day sodium selenite for eight weeks caused a substantial increase in cytotoxic T cells and Natural Killer (NK) cells by upregulating the rate of cell proliferation and differentiation into cytotoxic cells [105]. The same outcomes should be expected if selenite is supplemented naturally, i.e., via crop-food consumption.

Most of Se in the diet includes Se-containing amino acids (SeCys, SeMet, MeSeMet, and MeSeCys), which are essential for human health by acting as either direct and indirect antioxidants [32]. Compounds in which the Se moiety is methylated are the most powerful chemopreventive Se organic agents [33]. MeSeCys, in particular, by the action of cysteine conjugate β-lyase or related lyases, represents a reservoir of methylselenenic acid, a very potent agent versus cancer cell proliferation at very low concentrations. SeMet is, however, the main organic form of Se in plants, while SeCys is dominant in animal based-food [106]. Se supplemented in the form of SeMet to the human diet was reported to promote the synthesis of defensive proteins and antioxidant enzymes present on the mucosal surface, which is a physical barrier that prevents the entry of pathogens into the body [107]. After intake, SeMet is absorbed in the intestine through transporters for methionine and can then be metabolized in two different ways; it either directly participates in the protein synthesis, or is converted to SeCys, which is further metabolized with the production of selenide. SeCys, instead, is taken up by intestine cells using dibasic amino acid transporters and can operate as a direct antioxidant or be converted to selenide (Se^2−^). Hydrogen selenide ions (HSe^−^) can be used to form selenophosphate, which serves as a substrate for the synthesis of selenoproteins.

Se-amino acids are more promptly oxidized than analogs S-amino acids, and behave as strong nucleophiles [108]. Additionally, at pH close to neutrality, SeCys residues (free or in proteins) are mostly ionized; therefore, they manifest higher reactivity compared to Cys. This strong reactivity is a significant reason why SeCys is at the active site of mammalian selenoproteins of high redox activity. Concerning their direct role in antioxidant systems, Se-amino acids function by scavenging electrons from oxidants, complexing metal ions, and are quickly recycled after oxidation.

Other important Se organic metabolites that can be formed in the human body through high Se intake are low molecular weight compounds, including methylseleninic acid (a precursor of methylselenol) and many intermediates of selenium metabolism, including selenodiglutathione, dimethyldiselenide, and SeNPs, all possessing strong redox activity [109]. Similar to selenite, many of these Se compounds (e.g., methylselenol, dimethyl selenides, and SeNPs) prove to be effective against COVID-19 [10]. In contrast, this role is only hypothesized for other compounds [10]. These low molecular size Se species can induce modification of cysteine 145 residues of SARS-CoV-2 M (membrane) glycoprotein [10], which is one of the major membrane proteins of coronaviruses together with the spike and the envelope proteins [110], thus blocking the virus capacity to replicate. Additionally, volatile methylselenol has been postulated to be sequestered in the form of non-volatile methylseleninic acid in cells containing high levels of oxidants, such as those infected by SARS-CoV-2 or other viruses causing respiratory infections [111]. Methylseleninic acid in these cells can inactivate SARS-CoV-2 M protein (Mpro), thus reducing the inflammatory response to COVID-19.

In addition to inorganic and organic Se compounds of plant (and animal) origin, SeNPs generated through phyto- or phyco- synthesis could play a role against COVID-19 infection [95]. SeNPs are significantly effective agents against cancer and Huntington’s disease due to their antioxidant and immunity-induced effects [94]. Still, they could additionally be engaged as antiviral drug carriers, or loaded with antibiotics to contrast secondary bacterial infections that might develop following a SARS-CoV-2 attack to the organism. Recently, the antibacterial action of photosynthesized Se-NPs from *Spirulina platensis* extracts has been reported [112]. The advantage of using SeNPs in medical treatment compared to other forms of Se is associated with their high biocompatibility and chemical stability and low toxicity [94]. Furthermore, SeNPs are naturally degraded in the human body. Thus, SeNPs can exhibit equivalent or superior antiviral capability compared to inorganic and organic Se. Their use could overcome the issue of Se toxicity that might arise when high Se dosages are applied to fight against viruses.

It must be noted that, beyond natural Se-compounds, in many studies where Se was tested for its medical outcomes, synthetic Se compounds such as Ebselen (2-phenyl-1,2-benzoselenazol-3-one) were assayed. Ebselen, especially, was proven to be very effective in fighting COVID-19 and other respiratory viral infections by attenuating the concentration of inflammatory oxidants and cytokines, inhibiting Mpro, and reducing lung inflammation [113].

### 3.2. Plant Se Compounds Are a Source for Selenoproteins Synthesis

While Se is found in all the bioactive compounds as mentioned above, most of its essential biological functions are mediated by its integral constituent in selenoproteins. Thus, in the further sections, we will review the mechanism through which selenoproteins are produced, their multiple roles in viral diseases, and their possible association with COVID-19 infection.

#### 3.2.1. Generation of Selenoproteins: The SeCys Insertion Machinery

Selenium is not essential to all living organisms. In plants, for instance, Se at high concentration may cause toxicity as the amino acid SeCys can be misincorporated in proteins, thus disrupting their folding and functionality [114]. Conversely, SeCys in humans and other mammals is co-translationally and specifically inserted at the catalytic site of essential selenoproteins through a multiplayer-based molecular apparatus [2,115] (Figure 2). SeCys is more nucleophilic compared to the analog Cys. Therefore, its incorporation into selenoproteins shapes their unique structure and biological functions by enhancing their catalytic efficiency and antioxidant roles to maintain the cellular redox homeostasis [2].

Due to its essentiality in mammalian metabolism, SeCys is hence acknowledged as the 21st proteinogenic amino acid [116,117]. SeCys is introduced in the human body, especially via animal food, but is also produced from selenite and SeMet obtained from food crops. Most of SeCys is then converted to selenide, which is activated in metabolism by binding to phosphate [2].

The UGA codon, which is usually recognized as a termination codon for mRNA translation in other proteins, signals SeCys insertion in selenoproteins. The recoding of the UGA codon as a sense codon, also termed as Sec codon, is dictated by a cis-acting SeCys-insertion sequence (SECIS element) with a stem-loop structure. It is set downstream of the Sec codon in the 3′ untranslated region of selenoprotein mRNAs in eukaryotes [118]. Specialized trans-acting protein factors assist the deciphering of the Sec UGA codon, such as SBP2 (SECIS-binding protein), efSEC (an elongation factor specific to selenocysteine), and a selenocysteyl-specific tRNA (Sec tRNA[Ser]Sec) [119]. Prokaryotes possess homologs of all these factors, but the SECIS element localizes at a different position, i.e., proximal to the UGA codon [120].

Specifically, SeCys is synthesized from serine (Ser) on its own tRNA. Initially, the aminoacylation of tRNA^[Ser]Sec^ with Ser is catalyzed by seryl-tRNA synthetase (SerRS), which generates seryl-tRNA^[Ser]Sec^ [2]. This step is critical, as the Ser moiety provides the backbone to build up SeCys. Seryl-tRNA^[Ser]Sec^ is further converted to Sec-tRNA by selenocysteine synthase (SecS) through the formation of the intermediate O-phosphoseryl-tRNA^[Ser]Sec^. This process uses selenophosphate as a substrate, i.e., the active form of Se synthesized by selenophosphate synthetase (SPS) from selenide and ATP [121]. Thus far, two SPS proteins have been identified in humans based on homology with SelD from *Escherichia Coli*. Still, only SPS2, which is a selenoprotein itself, seems to carry out the de novo synthesis of selenophosphate, as corroborated by in vitro tests and complementation studies of SelD-deficient bacterial cells [122]. SPS2 also likely operates as an autoregulator of selenoprotein synthesis, whereas SPS1 is hypothesized to have a role in SeCys recycling and perhaps in other pathways unrelated to selenoprotein synthesis.

Upon Se shortage, SPS2 can use sulfide in place of selenide to produce thiophosphate as a substrate for SecS [122]. Thus, the final product from thiophosphate and O-phosphoseryl-tRNA^[Ser]Sec^ is Cys-tRNA^[Ser]Sec^, with Cys added at the catalytic site of selenoproteins at the UGA-encoded SeCys position in place of SeCys. The substitution of SeCys with other amino acids beyond Cys has been additionally reported. However, this event is ascribed to translational errors [2] occurring under prolonged treatment with antibiotics.

The SeCys machinery implies a series of steps to produce essential selenoproteins. When the selenoprotein mRNA is translated on the ribosome, the SECIS element drives the recoding of the in-frame UGA codons while preventing premature termination. Sec-tRNA^[Ser]Sec^, which possesses an anticodon complementary to the UGA, translates UGA as SeCys. SBP2, a protein steadily associated with ribosomes, specifically binds the SECIS element explicitly and interacts with eEFSec to promote the recruitment of Sec-tRNA^[Ser]Sec^ and SeCys incorporation in proteins [123].

The genes encoding mammalian selenoproteins were discovered by identifying the marker SECIS element downstream of in-frame UGA codons using computational approaches [124]. Although many selenoproteins have unknown functions, those that are well-characterized display unique antioxidant properties. The insufficient synthesis of antioxidant selenoproteins upon Se deficit might result in several pathophysiological disorders and, in some instances, in fatal diseases. The existence of a SeCys lyase that catalyzes the PLP(pyridoxal phosphate)-dependent degradation of SeCys to alanine and elemental Se in human cells might be essential for Se recycling and selenoprotein synthesis under Se deficiency [125]. It could be a potential target of therapeutic approaches. However, the function of this enzyme still requires a deeper investigation.

#### 3.2.2. Roles of Selenoproteins in Viral Diseases

In the scenario of SARS-CoV-2 spreading, plant Se compounds serving as sources of Se for the synthesis of selenoproteins relevant against viral diseases have attained increasing interest. Selenoproteins work coordinately to ensure the proper functioning of the immune system versus infections and inflammation conditions [9,15,24,28]. Among selenoproteins, it is worth mentioning glutathione peroxidases (GPx1, GPx2, GPx3, and GPx 4), thioredoxin reductases (TXNRD1, TXNRD2, and TXNRD3), methionine sulfoxide reductase B1 (MSRB1), selenoprotein P, selenoprotein K, selenoprotein W, peroxisome proliferator-activated nuclear receptor-γ, and IκB-kinase β ([10], references therein). Such proteins can act through different mechanisms for counteracting pathogens [126]. For instance, they display antioxidant (e.g., GPx and TXNRD), redox (e.g., TXNRD), anti-inflammatory (e.g., GPXs, TXNRDs, and selenoprotein S), and immune functions (e.g., GPxs, TXNRDs, MSRB1, selenoprotein K, and selenoprotein S) [126].

Individuals with low Se dietary intake are likely to be more vulnerable to virusattacks due to an increased generation of intracellular ROS resulting from selenoprotein depletion [28] and SARS-CoV-2 interference with the selenoprotein array [10]. Interestingly, Moghaddam et al. [127] found that Se status, evaluated in terms of Se concentration in plasma and SelP expression, was substantially higher in surviving SARS-CoV-2 patients than in non-survivors. In line with this study, a positive correlation between Se status and prognosis of SARS-CoV-2 infection was established among individuals affected by this virus in China [128]. In Enshi, which is one of the areas in the world where the local population has the highest Se intake in the diet, the recovery rate from COVID-19 was almost triple the average for the other cities in Hubei Province [129]. Additionally, other very recent studies provide evidence of a potential link between SARS-CoV-2 inflammation and the Se status, as infected cells in culture contained reduced amounts of selenoproteins and concurrently developed the production of factors (e.g., cytokines) that deplete their biosynthesis [126]. In particular, increased expression of IL-6, an inflammatory cytokine positively correlated with the severity of SARS-CoV-2 [126], was reported to decline the concentration of SelP and expression of deiodinase type 1 (DIO1) [130]. Hypercytokinemia (i.e., high levels of cytokinins) observed in hosts affected by severe SARS-CoV-2 infection is responsible for inducing damage to the lung epithelium cells that can promote the occurrence of further infections, either bacterial or fungal [12].

SARS-CoV-2 also overwhelms the transcription of selenoproteins associated with ferroptosis (GPx4), endoplasmic reticulum (ER), stress (selenoprotein F, selenoprotein K, selenoprotein M, and selenoprotein S), and DNA synthesis (thioredoxin reductase 3) [126]. The decrease in ER-associated selenoproteins can cause protein misfolding and enhance ER stress, with detrimental outcomes in cells [126,131].

The mechanism through which SARS-CoV-2 causes depletion of selenoproteins might be the same reported for other RNA viruses, such as HIV-1 and the Zaire strain of the Ebola virus (EBOV). These viruses determine the knockdown of selenoproteins, such as thioredoxin reductases, via RNA: RNA antisense interactions, thus increasing the pool of ribonucleotides for viral RNA synthesis while repressing DNA synthesis [132]. Decreased antioxidant activity due to SARS-CoV-2-induced repression of selenoproteins in cells may result in a weakened immune system and induction of genome mutations in viruses that convert them to highly pathogenic strains [133,134,135,136,137]. This process was at least reported for coxsackievirus B3 (CVB3), influenza virus type A /Bangkok/1/79 (H3N2), influenza H1N1, human immunodeficiency virus/acquired immunodeficiency syndrome (HIV/AIDS), polio, hepatitis B and C, and hantavirus [28]. Keshan disease, for instance, arises when CVB3 mutates to a more virulent strain in a host deficient in Se [87,107,138,139]. Unfortunately, RNA viral mutations are reported to be faster, more frequent, and long-lasting in Se-flawed hosts [140].

Therefore, Se-enriched food crops should be provided to people with suboptimal Se in their diet; this would lower the severity of COVID-19 detrimental effects, virus mutation frequency, and replication by providing adequate Se to synthesize selenoproteins. Though Se impact is sometimes evident at supraoptimal levels, i.e., higher than the recommended dose (e.g., in the case of selenite and MetSeCys), more studies need to be done to definitively ascertain how Se intake can represent a crucial factor in determining the intensity of the host response to SARS-CoV-2, and which factors could limit the benefical effect of Se species. For instance, chronical exposure to low levels of toxic metals, as it may happen in heavily polluted areas, can impair the Se human metabolism due to declined concentration of certain selenoproteins in blood plasma [141]. This event may potentially reduce the protective role of dietary Se compounds against COVID-19 infection. In support of this hypothesis, one may recall that the high COVID-19 related death rate near Bergamo (Italy) in April of 2020 could be rationalized by the fact that this town is located in a region (Lombardia) that suffers from the highest air pollution in all of Europe.

## 4. Recent Case Studies Ascertaining the Link between Se Status and Resilience to COVID-19

A variety of factors including age, physiological and nutritional status, immunity strength, and genetic and environmental factors appear to be important determinants of the susceptibility of individuals to SARS-CoV-2 infection and disease severity [142]. Se supplementation effectively mitigates moderate and severe infections caused by different viruses [28] due to the high redox properties of Se compounds and the functions of selenoproteins in the host immunity. Thus, the association of Se deficiency with the severity of COVID-19 disease is not surprising.

Low or suboptimal Se intake in the diet is related to increased oxidative stress and hyper inflammation, mainly due to depletion of ROS scavenging and anti-inflammatory selenoproteins [28]. People with low Se in the plasma serum are likely to be more susceptible to COVID-19 disease and, possibly, to the high frequency of SARS-CoV-2 mutations. On the other hand, individuals with adequate levels of Se in the organism are expected to be more resilient to the detrimental effects of COVID-19.

Thus far, a few recent investigations have provided strong evidence for a nexus between Se status and COVID-19 outcomes [142,143]. Most of these studies were conducted in China, as this country has populations with both the lowest and the highest Se status globally. In particular, Zhang et al. [128] compared cure rates and death rates in Hubei province and outside. The survey revealed that inside Hubei province, the cure rate determined in Enshi city was about three times higher (36.4%) than in other cities (mean 13.1%). This was likely as the Se status of the Enshi population is notoriously very high according to the elevated Se intake (550 µg/d measured in 2013) [129]. However, the cure rate in Hubei province was lower compared to other provinces (13.2% vs. 40.6%), and the death rate was concomitantly higher. A possible explanation is that in Hubei province, which is the site the COVID-19 disease initially broke out, the fatality rate was strongly affected by the inadequacy of medical and health cures at the early stages of the first wave of the COVID-19 epidemic.

Zhang et al. [143] evaluated the association between COVID-19 related fatality and the Se content in crops and topsoil at the population level in China in one year (December 2019–2020). The authors considered 14,045 COVID-19 cases reported from 147 cities, and found that the case fatality rates (CFRs, calculated by dividing the observed number of deaths by the number of confirmed cases) increased from 1.17% in Se-sufficient areas (>0.06 ppm Se) to 3.16% in highly deficient Se areas (<0.03 ppm). Concurrently, when correlations were estimated between CFRs and Se content in topsoil, the CFRs values were 0.76% and 1.85% in Se normal and low Se areas, respectively. The fatality risk was more pronounced in cities where inhabitants fed on low Se crops vs. cities where consumers had adequate Se dietary intake, with the incidence rate ratio (IRR) equal to 3.88 (patients per 1000). Hubei province and cities with only a few cases and cases imported from abroad (out of China) were not included in the survey.

In the study conducted in Germany by Moghaddam et al. [127], Se in the serum of 33 ill COVID-19 patients (166 samples analyzed in total) was found to be definitely low (mean ± SD, 50.8 ± 15.7 vs. 84.4 ± 23.4 µg/L of the reference population) to ensure the proper synthesis of selenoproteins, especially SelP (3.0 ± 1.4 vs. 4.3 ± 1.0 mg/L of the reference population). Conversely, the Se status of individuals that recovered from COVID-19 was substantially higher compared to non-survivors. The authors claimed that the analysis of Se status in COVID-19 infected patients has diagnostic relevance based on their findings. They also confirmed the critical role of Se during post-infection, and encouraged the administration of Se as adjuvant against COVID-19 adverse outcomes.

## 5. Conclusions

A large proportion of the world population has inadequate selenium intake, which has relevant consequences on COVID-19 infection spreading and the relative outcomes. Recent studies support the existence of a strong association between the low Se status and the severity of COVID-19 disease. Indeed, Se in both inorganic and organic form exhibits remarkable positive effects in the human body, and is required to synthesize essential selenoproteins with antioxidant and immune properties. In individuals that are Se deficient, the amount of selenoproteins is, however, reduced.

Most Se in the diet is gained from crop food. Therefore, increasing the Se nutritional status of vulnerable populations can represent a valuable tool to naturally enhance their immune system to better contrast viral disease, including that caused by SARS-CoV-2. On this account, biofortification programs aimed at increasing Se accumulation by crops in forms that might benefit the organism attain increasing relevance in the present scenario, especially in those countries where Se in soil and crops is relatively low.
